# Immunological Roles of NLR in Allergic Diseases and Its Underlying Mechanisms

**DOI:** 10.3390/ijms22041507

**Published:** 2021-02-03

**Authors:** Miranda Sin-Man Tsang, Tianheng Hou, Ben Chung-Lap Chan, Chun Kwok Wong

**Affiliations:** 1 Department of Chemical Pathology, The Chinese University of Hong Kong, Hong Kong, China; sinmantsang@cuhk.edu.hk (M.S.-M.T.); 1155118841@link.cuhk.edu.hk (T.H.); 2 State Key Laboratory of Research on Bioactivities and Clinical Applications of Medicinal Plants, Institute of Chinese Medicine, The Chinese University of Hong Kong, Hong Kong, China; benchan99@cuhk.edu.hk; 3 Li Dak Sum Yip Yio Chin R & D Centre for Chinese Medicine, The Chinese University of Hong Kong, Hong Kong, China

**Keywords:** allergy, inflammasome, molecular mechanism, NOD-like receptors, NLR

## Abstract

Our understanding on the immunological roles of pathogen recognition in innate immunity has vastly increased over the past 20 years. Nucleotide-binding oligomerization domain (NOD)-like receptors (NLR) are cytosolic pattern recognition receptors (PRR) that are responsible for sensing microbial motifs and endogenous damage signals in mammalian cytosol for immune surveillance and host defense. The accumulating discoveries on these NLR sensors in allergic diseases suggest that the pathogenesis of allergic diseases may not be confined to the adaptive immune response. Therapy targeting NLR in murine models also shields light on its potential in the treatment of allergies in man. In this review, we herein summarize the recent understanding of the role of NLR sensors and their molecular mechanisms involved in allergic inflammation, including atopic dermatitis and allergic asthma.

## 1. Introduction

Allergy is a chronic inflammatory condition triggered by allergens. Atopic dermatitis (AD) clinically characterized by pruritus, lichenification, reddening and dryness of the skin, and allergic asthma, featuring the presence of coughing, wheezing, tightness in the chest and shortness of breath, are two common allergic diseases found in children. The skin barrier and epithelial barrier of the lung not only provide a site of the entry for allergens, but also a route for pathogen invasion. Microbial and viral infections are common contributors to the symptoms and exacerbation in AD and allergic asthma. Gene–gene interaction, immune dysregulation as well as environmental factors are the keys in the pathogenesis of these two allergic diseases.

Innate immunity is the front line of defense against pathogens and dangers that is conserved in animals and plants. To initiate a rapid host defense and response against microbial infections, cellular damage and stress, intracellular and extracellular pattern recognition receptors (PRRs) will first recognize microbial signatures including Pathogen-Associated Molecular Patterns (PAMP), endogenous damage molecules Damage-Associated Molecular Patterns (DAMP), as well as stress signals. Unlike the adaptive immunity, this rapid recognition of pathogens and dangers by the germline-encoded PRRs does not require any priming or memory against pathogens that the cells encountered previously, but the highly conserved microbial patterns are vital for the survival of the microorganisms.

In response to the stimuli, the activated cytosolic PRRs will trigger protease caspase-1 to initiate the maturation and the subsequent release of inflammatory cytokines interleukin-1 (IL-1) and IL-18 or the induction of pyroptosis, which is a caspase 1-dependent programmed cell death that involves a rapid plasma-membrane rupture and pro-inflammatory content release of the microbial-infected or damaged cells [1]. These downstream inflammatory events ultimately facilitate the elimination of the invading pathogens; hence, they resolve infection and protect the host from disease development [2].

### 1.1. NLR Family

Major PRR families identified in mammals include Toll-like receptors (TLRs), nucleotide-binding leucine-rich repeat-containing proteins/nucleotide-binding oligomerization domain (NOD)-like receptors (NLRs), retinoic acid inducible gene I (RIG-I)-like receptors (RLRs), absent in melanoma 2 (AIM2)-like receptors (ALRs), C-type lectin receptors (CLRs), pyrin and intracellular DNA sensors. Unlike the well-known PPR family TLRs and CLRs, which are transmembrane receptors that sense extracellular and endosomal microbes, NLRs belong to another large PPR family that is present in the cytosols and is responsible for non-specific recognition of intracellular microbial products, including peptidoglycan, which is a molecular complex present in all bacteria cell walls [3]. Moreover, NLR also serves as the backup host defense system against invaded pathogens and in tissues where other PRRs are compartmentalized and acts jointly with other PRRs, especially TLR, to generate a substantial innate immune response [4].

**Figure 1:**
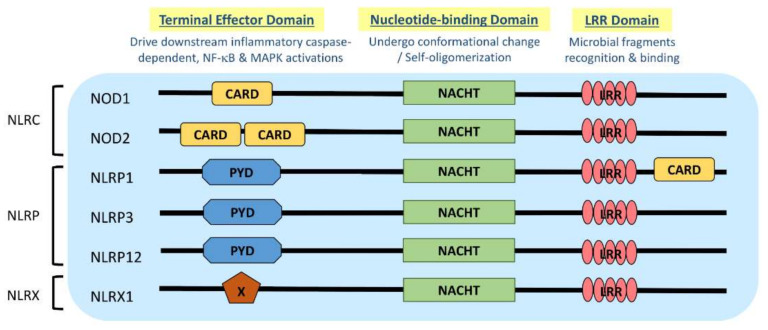
Domain architecture of NOD-like receptor (NLR): NOD1, NOD2, NLRP1, NLRP3, NLRP12 and NLRX1. NLR genes encode the intracellular multidomain proteins NLR, which are composed of a leucine-rich repeats (LRR) domain in the C terminal, the intermediate nucleotide-binding NACHT domain and a terminal effector domain in the N terminal. The variables caspase activation and recruitment domain (CARD), pyrin domain (PYD) and undefined domain (X) in the terminal effector domain further divide NLR into subfamilies, NLRC, NLRP and NLRX, respectively. Gene polymorphisms and gain-of-function mutations identified on these NLR genes in atopic dermatitis (AD) and allergic asthmatic patients may contribute to the aberrant expressions of NLR for microbial surveillance, asthma protection and exacerbation.

NLR is a protein structurally composed of three domains, namely (1) the microbial pattern recognition domain with leucine-rich repeats (LRR) located at the C terminus; (2) the nucleotide-binding NACHT-(NAIP (neural apoptosis inhibitory protein), CIITA (MHC Class II transactivator), HET-E (vegetative incompatibility protein from *Podospora anserina*) and TP-1 (telomerase-associated protein 1)) domain in the center that is essential for self-oligomerization, and (3) the terminal effector domain at the N terminus that drives downstream inflammatory caspase and NF-κB activation (Figure 1) [3]. There are so far around 22 NLR proteins identified, each carrying a distinct domain organization. Effector domains at the N terminus, which are crucial for signal transduction, help further divide these NLRs into five subfamilies, namely NLRA, NLRC, NLRC, NLRP and NLRX. Typical NLRs in the NLRC subfamily, which is characterized by the caspase activation and recruitment domain (CARD), include the nucleotide-binding oligomerization domain-containing protein 1 (NOD1) and NOD2; while the NLRP subfamily, which is characterized by the pyrin domain (PYD), includes NLRP3 (NOD-, LRR-, and pyrin domain-containing protein 3) (Figure 1) [3]. NLRC and NLRP are the two major subfamilies that are found to be relevant to allergic diseases (Table 1). In this review, the recent advances about the role and the underlying mechanisms of NLRs in these two NLR subfamilies in AD and allergic asthma will be discussed.

### 1.2. NLR and Atopy

Gene polymorphisms and haplotype combinations of NLR genes identified in atopic individuals revealed that NLR could be vital determinants of atopy susceptibility. NOD1 is located on the chromosome region 7q14-p15, which is a region believed to be atopy susceptible [6]. At least three of the polymorphisms located on the NOD and LLR domains of NOD1 gene are positively associated with the elevated total serum IgE level, while a haplotype on the NOD1 gene exerts a significant protective effect against IgE elevation in a German cohort [56]. Insertion polymorphism of the NOD2 gene may enhance the severity of atopic status, as it causes a 50% increase in risk of developing atopy and an elevated level in serum IgE in atopic children [57]. A Swedish cohort also revealed a significant association between the NLRP3 variant and total IgE antibody level exclusively in males [58]. The frameshift mutation may lead to truncation of the ligand-binding region LLR domain which then impairs the downstream NF-κB signaling upon bacterial lipopolysaccharide (LPS) stimulation [59]. Moreover, short isoform of NOD2/CARD15 was found to be an endogenous inhibitor of NOD2-induced NF-κB activation [60]. Impaired function of the innate immunity due to the CARD15 polymorphisms to recognize microbial exposure may give rise to Th2-dominant allergy [57]. The hygiene hypothesis suggested that early exposure to microbes can protect an individual from atopic disease by facilitating the development of the regulatory T cell (Treg) reservoir. Lack of the ability to recognize bacterial challenge may lead to the development of allergic diseases. It is also hypothesized that the NOD2 polymorphism affects the apoptosis of inflammatory cells, leading to a persistent and excessive inflammatory response as observed in atopic diseases, yet this hypothesis is yet to be proven [57].

## 2. NLR in Atopic Dermatitis

### 2.1. Gene Polymorphisms in NLR in AD

Genetic variations in NLR genes may contribute to the pathogenesis of AD. Single nucleotide polymorphisms (SNPs) in NOD1 (or CARD4), NOD2 (or CARD15) and NALP12 genes were identified in AD individuals in several German cohorts [57,61,62]. Moreover, interactions among polymorphisms in the CARD4 gene, as well as between the polymorphisms in the promoter of CARD12 and NALP1 genes (and a rare CARD4 haplotype) were also found in AD individuals [62]. A haplotype on NOD1 gene, on the other hand, was reported to be protective against AD in one of the German cohorts [61]. Although no associations were found between AD and the autoimmune disease/inflammatory-related SNPs on NLRP3 gene screened in the Brazilian and Swedish cohorts, the possibility of other undetected association between the unassessed SNPs with AD cannot be ruled out [58,63,64,65,66]. For example, several NOD2 gene polymorphisms were identified in patients with auto-inflammatory Yao syndrome, which presents with recurrent dermatitis and eyelid swelling like AD and allergic rhinitis, respectively [67,68], as well as NLRP3 and NLRP12 mutations in IgE-associated cold-induced urticaria [69,70] are potential NLR candidates that should be explored. In contrast to the Brazilian cohort, SNP (rs12150220) on NLRP1 gene was found to be associated with AD in the Swedish cohort [58,63]; while NLRP1 gene expression in the unaffected skin of AD individuals was also found to be negatively correlated with AD severity as reflected by EASI index [71], indicating the intrinsic genetic variation of NLRP1 could be AD-related.

Polymorphisms in NLR genes could alter the NLR protein expression, which in turn leads to a dysregulation in pathogen recognition and response in AD patients. The missense-coding SNPs located on the highly conserved inflammasome genes NLRP1 and NF-κB-inhibitor CARD8 found in Swedish AD individuals were predicted to be functionally significant in their susceptibility of AD [58]. Sparse wound healing and defense response in severe AD patients may be caused by the downregulation of NLRP1, making their skin more prone to adverse reactions upon viral infection [71]. While it is proposed that insufficient immune stimulation can be due to the lack of microbial contacts in individuals living in industrialized countries; polymorphisms in CARD15 may also explain the deficient immune stimulation observed in AD individuals [62].

Functional assays in NLR gene knock-out models can help to further confirm the pro-inflammatory role of the NLR genes in the pathogenesis of AD. The Nlrp12−/− mice exhibited an attenuated inflammatory response and reduced allergic dermatitis-like features, without affecting pro-inflammatory cytokines production (including IL-1β), antigen processing and presentation or T cell proliferation upon oxazolone-induced sensitization and challenge [1]. The migration capacities of bone marrow-derived dendritic cells (BMDC) from skin to the draining lymph nodes and neutrophils from the blood stream to the skin of the Nlrp12-deficient mice, however, were significantly reduced. These impaired migration capacities were not observed in another NLR-knockout Nlrp3−/− mice. Although the expressions of CCR7 and CXCR4 on Nlrp12−/− BMDC were unaffected, the response of BMDC towards their ligands CCL19, CCL21 and CXCL12, and the responses of Nlrp12−/− neutrophils towards neutrophils-chemoattractant CXCL1 were significantly reduced. These data indicate that NLRP12 promotes allergic skin inflammation via facilitating DC retention in the periphery and neutrophils migration but not inflammasome activation and IL-1β secretion [1].

### 2.2. NLR and S. aureus in AD

Colonization of Staphylococcus aureus (*S. aureus*) was found in approximately 80–100% of AD patients [40]. Barrier dysfunction, dryness, and increase in trans-epidermal water loss of the skin all provide an easy route for these colonized pathogens to invade [6]. As innate immunity is responsible for protecting the host from pathogens and initiating repair upon injury and damage; TLR2 has been recognized as the principal receptor for combating *S. aureus*. However, recent studies showed that intracellular NLRs also play a role in microbial pathogen recognition. Upon pathogens invasion, intracellular NLRs such as NOD1, NOD2, NLRP1, NLRP3, NLRC4 (NOD-, LRR- and CARD-containing 4) and interferon-inducible protein AIM2 detect the microbial motifs, danger signals or stimuli, and initiate a cascade of inflammatory response as anti-microbial response.

### 2.3. NOD1 and NOD2 in AD with S. aureus Colonization

**Figure 2:**
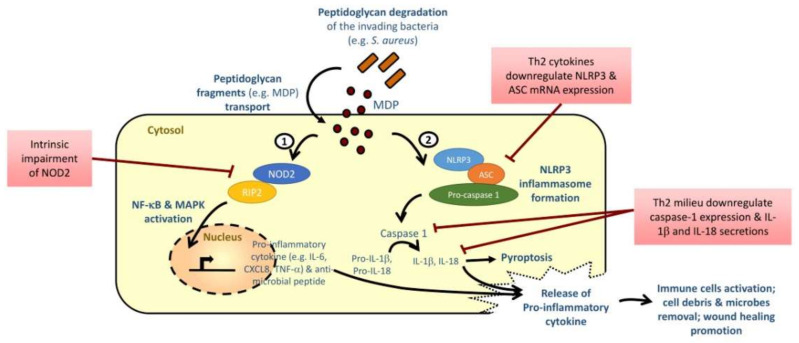
Th2-dominant milieu and impairment of NLR disrupt cutaneous defense against microbial pathogens in AD patients. *S. aureus* colonization is commonly observed in skin lesion of AD patients. Peptidoglycan fragments such as muramyl dipeptides (MDP) are constantly degraded from *S. aureus*, and are transported into the cells by bacterial secretion, endocytosis, membrane transport system or via outer membrane vesicles. MDP in the cytosol can then activate nuclear factor (NF)-kB and mitogen-activated protein kinase (MAPK) signaling, and trigger the release of downstream pro-inflammatory cytokines via (1) NOD2 recognition or (2) formation of NLRP3 inflammasome. NOD2, anti-microbial peptide and NLRP3 inflammasome components were found to be impaired or downregulated in basophils and keratinocytes of AD patients. Th2 cytokines also contribute to the suppression of its downstream caspase-1-dependent pro-inflammatory cytokine secretions. These abnormalities may explain their failure in microbial pathogen recognition, immune cell activation, wound healing and host defense.

Peptidoglycan is a major cell wall component conserved in all Gram-positive and Gram-negative bacteria, including *S. aureus*. It confers the cell shape and provides mechanical protection to the bacteria, and hence is inevitable in bacterial survival [6]. NOD1 and NOD2 can readily recognize the degradation products of peptidoglycans, i.e., diaminopimelic acid (DAP) and muramyl dipeptides (MDP), respectively, and such recognitions are essential in the early detection of PAMP of the host (Figure 2) [5,18]. The binding of ligands DAP and MAP to NOD1 and NOD2 will initiate a signal transduction cascade, which triggers the translocation of NF-κB to the nucleus. The NF-κB then induces the gene transcription of pro-inflammatory cytokines and anti-microbial peptides for host defense [6]. NOD can also synergize with TLR and the associated activation signaling pathways and initiate potent antimicrobial immune response [6,7,17].

The role of NOD1 in the pathogenesis of AD is largely unknown, but NOD2 is recognized as a contributor in cutaneous defense against *S. aureus* in innate inflammatory response [72]. Expression of NOD2 on circulating basophils, one of the initiators and accessory cells for Th2 polarization in allergic inflammation, was found to be significantly lower in AD patients than in healthy individuals [73]. Stimulation with *S. aureus*-associated NOD2 ligand MDP can induce the release of inflammatory cytokine IL-6 and chemokine CXCL8 via activating mitogen-activated protein kinase (MAPK)/extracellular signal-regulated kinase (ERK) and NF-κB pathways in the co-culture of human dermal fibroblasts and human eosinophils, which are the principal effector cells in allergic inflammation commonly found in the skin biopsies of AD patients, but such stimulating effects were not observed in human basophils [16]. By utilizing an MC903-induced AD-like murine model, our group further confirmed the interaction of *S. aureus* with NOD2 on eosinophils but not on basophils in the exacerbation of skin inflammation; however, expansion of basophils in vivo could restore the exacerbation effect induced by NOD2 ligand MDP [16]. Moreover, MDP induce lower levels of tumor necrosis factor-α (TNF-α), IL-6 and CXCL8 from peripheral blood mononuclear cells (PBMCs) of AD patients when compared to healthy controls ex vivo [73]. The above studies indicate that the recognition of *S. aureus* involves NOD2; however, NOD2-mediated innate immunity of basophils and PBMCs may be impaired in AD patients [73]. The downregulated innate immune mechanisms may facilitate the proliferation of *S. aureus* in the skin lesion of patients with AD, and subsequently lead to the uncontrolled stimulation of other leukocytes in the circulation [73].

Peptidoglycan-derived MDP are the minimal bacterial structure recognized by NOD2 [15,18]. Peptidoglycans degradation during the bacterial life cycles will constantly lead to the formation and release of peptidoglycan fragments (or muropeptides) (Figure 2) [4,74]. These peptidoglycans fragments are then transported into the cytosol of eukaryotic cells through bacterial secretion systems, endocytosis or membrane transport systems or are delivered via outer membrane vesicles [74]. Direct or indirect binding of ligand to the LLR domain will trigger the conformational change of the NLR, exposing the NACHT domain to mediate oligomerization and the exposure of effector N terminal [4]. The subsequent recruitment of receptor-interacting serine/threonine-protein kinase (RIPK2) then initiates the activation of NF-κB and MAPK pathways [75]. MDP can also induce the release of anti-microbial peptide β-defensin-2 via NOD2 in keratinocytes [19]. Such β-defensin-2 is also the linker between the innate and adaptive immunity by recruiting dendritic cells and T cells to the site of microbial invasion [76]; but its expression level is significantly decreased in acute and chronic lesions of AD patients [77]. Impairment of NOD2 in AD is therefore hypothesized to result in perturbed bacterial recognition, and the subsequent insufficient downregulation of immune response for guarding host/bacterial interactions as observed in other chronic diseases, such as Crohn’s disease [78], as well as failure in bacterial clearance in AD skin lesion.

### 2.4. NLRP3 in AD with S. aureus Colonization

NLRP3 can normally form a tripartite protein complex inflammasome with the adaptor protein apoptosis-associated speck-like protein (ASC) and an inactive zymogen pro-caspase-1 (Figure 2). The inflammasome assembled will induce the processing of effector protein caspase-1, which subsequently cleave the inactive precursors pro-IL-1β and pro-IL-18 into pro-inflammatory cytokines IL-1β and IL-18 [31]. These pro-inflammatory cytokines can then activate monocytes, macrophages, neutrophils to phagocytose the invading pathogens, remove cellular debris and microbes, induce adaptive Th1 and Th17 response for host defense, stimulate wound healing and restore homeostasis [79]. MDP from the bacterial peptidoglycans, but not LPS, was found to induce NLRP3 inflammasome, activate caspase-1, and trigger maturation of pro-IL-1β [20]. NC/Nga mice kept under conventional conditions developed AD symptoms with a significantly higher skin NLRP3 expression than those maintained under specific pathogen-free (SPF) conditions, which showed no AD symptoms [37], indicating that pathogenic environment may induce NLRP3 expression and subsequently trigger AD development. Ultraviolet B eye irradiation could further induce NLRP3 expression and aggravate AD inflammation in this spontaneous AD mice model [37]. While mice with the overexpression of casepase-1 in keratinocytes will spontaneously develop dermatitis with high serum IL-18 level [80,81]; treatment with caspase-1 inhibitor or blocking IL-18, however, significantly mitigate the AD symptoms and reduce plasma thymic stromal lymphopoietin (TSLP) level in AD mice with or without UVB irradiation [37], which further implicated that NLRP3 inflammasome and its downstream players caspase-1 and IL-18 are paramount in both development and exacerbation of AD. IL-18 level was found to be elevated in the serum of AD patients and NC/Nga mice before the onset or during the development of AD [82]; it could contribute to the Th2 response and was suggested to be a biomarker to assess the severity of AD [38,41,42,43,44]. IL-1β level, on the other hand, was significantly elevated in the skin lesion of AD patients with filaggrin gene (FLG) mutation [83]. However, a study also showed that inflammasome in skin could inhibit Th2-related epidermal TSLP expression in AD mice when the IL-1 signaling is intact [84].

Although no genetic predispositions on NLRP3 have been discovered in AD individuals, NLRP3 and its effector protein caspase-1 were impaired in the lesional skin of AD patients, when compared to healthy controls [40]. Interestingly, unlike Th1 cytokines, allergy-related Th2 cytokines can downregulate the mRNA expression of NLRP3 and ASC in primary keratinocytes (HPK) [40]. The same study also revealed that Th2 milieu can downregulate caspase-1 and suppress the downstream secretion of IL-1β from the primed monocytes of AD patients upon the trigger of an inflammasome activator staphylococcal exotoxin α-toxin. The downregulation of these NLRP3 inflammasome components, which may be important for *S. aureus* recognition, were suggested to be induced by the presence of IL-4, IL-5 and IL-13 in AD patients [40]. Clinical manifestations of microbial infection, such as impetigo contagiosa and paronychias, are commonly observed in the skin of AD patients; and the presence of *S. aureus* colonization even on the non-lesional skin of over 50% of the AD patients [6]. It indicated that there is a possible failure in these innate defense mechanisms against the invading bacteria. Niebuhr et al. also suggested that failure in *S. aureus* recognition and the downstream pro-inflammatory cytokine secretion may explain the *S. aureus* infections as observed in AD patients [40]. It is speculated that bacterial infection caused by the impaired NLR function contributes to skin inflammation in AD patients.

### 2.5. NLRP3 Inflammasome and Commensals Fungal and Yeast in AD

Yeast and fungus are other major types of colonizing microbes commonly found on the lesional skin of AD patients [85]. Although the etiological role of these superficial commensals such as the Malassezia spp. on the skin lesion of AD patients remains controversial, the involvement of PRR in the anti-fungal response has been widely reported [86,87]. Fungal cell wall component β-glucan was able to activate NLRP3 inflammasome via a potassium efflux pathway in macrophages [39]. *Candida albicans* and *Aspergillus fumigatus* were able to activate NLRP3 inflammasome, and hence induce the secretion of pro-inflammatory cytokine IL-1β via a spleen tyrosine kinase (Syk)-dependent pathway [34,35,36]. Human monocytes and monocyte-derived dendritic cells primed with ultra-pure lipopolysaccharides (upLPS) were reported to sense heat-resistant factors from Malassezia spp., activate NLRP3 inflammasome and subsequently secrete a robust level of pro-inflammatory cytokine IL-1β via a caspase-1-dependent pathway and the C-type lectin receptor (CLR) in seborrheic dermatitis [88]. Therefore, fungal-induced NLRP3 inflammasome contributes to skin disease onset and progression via inflammatory cytokine production and cell death pathways [89], yet the involvement of NLR in this induction remains to be elucidated.

## 3. NLR in Allergic Asthma

### 3.1. NLR and the Hygiene Hypothesis in Allergic Asthma Protection

It has long been postulated that microbes play a regulatory role in allergic asthma development. In line with the hygiene hypothesis, which states that adequate microbial exposure in early life protects individuals from developing allergy development including asthma, high dose of LPS stimulation can inhibit the production of specific IgE in mice [90]. Computational modeling revealed that NLR member NLRX1 in macrophages can also modulate innate immunity against *Helicobacter pylori* (*H. pylori*) infection [53]. Exposure to gastric bacteria *H. pylori* can also drive asthma protection by inducing IL-18 in dendritic cells through the NLRP3/CASP1/IL-18 axis [54,55].

The SNP on NOD1 identified in children was found to be associated with asthma protection in a European cohort [90]. It is speculated that immune response against NOD1 ligand DAP was amplified in individuals who carry this SNP on NOD1 under an environment of a high level of endotoxin [90]. Nevertheless, another genetic polymorphism of NOD1 was also identified in asthmatic individuals [91]. Such spliced NOD1 variants could affect DAP recognition, and hence cause potential interruption of the downstream NF-κB pathway that is normally triggered by the full-length NOD1 molecule [13]. Insufficient priming to a diverse group of commensal bacteria in early childhood can lead to the loss of immunological tolerance establishment, which has been linked to the asthma susceptibility [92]. Dysregulated expression of the truncated NOD1 isoform upon inflammatory stimulations could ultimately contribute to different disease states in asthma development [13].

However, bacterial infection is, at the same time, a risk factor of asthma development and exacerbation [93]. Pulmonary bacterial infection *Chlamydia pneumonia* can trigger allergic airway sensitization and eosinophilic inflammation towards inhaled allergens through dendritic cell activation [93,94]; meanwhile, mycoplasma can cause asthma exacerbation [95]. Certain commensal bacteria can also aggravate inflammation in an asthmatic mice model via the NLRP3/IL-1β axis [45]. NOD2 ligand and NLRP3 inflammasome can also promote Th2 polarization, resulting in the susceptibility to inflammation in the asthmatic lung of mice [22,46]. The allergen house dust mite (HDM) can activate RIP2, a downstream player in NOD stimulation [10]; it can also elicit pyroptosis in bronchial epithelial cells via NLRP3 inflammasome [50]. Yet, the loss of RIP2 does not affect the group 2 innate lymphoid cells (ILC2) in the lung of an HDM-induced allergic asthmatic model [10], the involvement of ILC2 in NOD stimulation remains to be elucidated. NLRP3 inflammasome can upregulate IL-4 to promote M2 macrophage polarization, which are cells that contribute to the inflammation in allergic asthma [49]. A report, however, showed that NLRP3 is not required in the regulation of eosinophils in allergic airway inflammation [44]. Activation of caspase-1 even dampens IL-33-dependent allergic asthmatic inflammation [47].The involvement of NLRP3 is also reported in neutrophilic asthma, another type of asthma that is often associated with non-allergic exposure [96,97]. Particulate matters, ozone and apoliopoprotein E can aggravate asthma via NLRP3 inflammasome [98,99,100,101]. Different agents have been suggested as potential targets in alleviating hyperresponsiveness and inflammation in allergic asthmatic models by suppressing NLRP3 inflammasome activation [102,103]. Although the involvement of NLRP3 inflammasome in asthma pathogenesis has been widely reported, such an allergic inflammatory response is mainly triggered by the TLR-pathways in murine models and humans [55,94,104,105,106,107]. On the other hand, gain-of-function SNPs in NLRP1 was recently reported to activate inflammasome and hence, trigger asthma development and exacerbation [30]. Collectively, the specific roles of NLRP1 and NLRP3 in allergic asthma are still unclear.

### 3.2. NLR of Eosinophils and Bacterial Infection in Allergic Asthma

**Figure 3:**
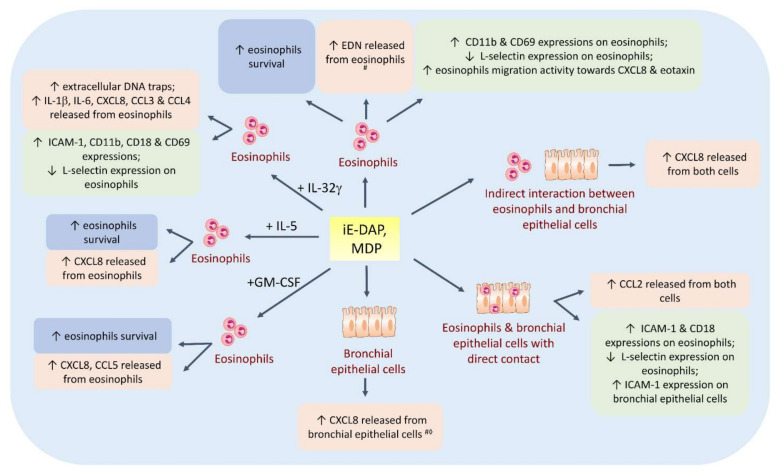
Effects of NLR ligands iE-DAP and MDP on human eosinophils, bronchial epithelial cells and their co-culture in vitro. NLR ligands promote eosinophil survival, downregulate L-selectin expression on eosinophils, but upregulate adhesion molecule ICAM-1 on eosinophils and bronchial epithelial cells with or without IL-32γ, IL-5 or GM-CSF treatment or in co-culture system; thus, they facilitate cell adhesion, chemotactic and migration of leucocytes. NLR ligands also induce the release of pro-inflammatory cytokines IL-1b, IL-6, CXCL8, CCL2, CCL3, CCL4 and CCL5 from eosinophils and bronchial epithelial cells to trigger inflammation in the lung. ^#^ The increase in eosinophil-derived neurotoxin (EDN) released from eosinophils/CXCL8 released from bronchial epithelial cells is observed upon MDP stimulation only. ^◊^ Results varied across publications.

In fact, NLR also constitute a link with respiratory infections, and play a key role in modulating allergic asthmatic inflammation. NOD1 and NOD2 are constitutively expressed and can be induced in human bronchial epithelial cell line BEAS-2B cells [8,23]. Functionally active NOD1 and NOD2, but not NALP3, are also expressed in human eosinophils, which are the terminal effector cells in allergic asthma that release tissue-damaging mediators in the inflammatory lung [11]. Interestingly, stimulation with the NOD1 ligand γ-D-glutamyl-meso-diaminopimelic acid (iE-DAP) or NOD2 ligand MDP alone could not consistently induce the release of pro-inflammatory cytokines and upregulate the adhesion molecules or activation marker of human eosinophils or BEAS-2B cells (Figure 3) [9,11,14]. Treatment with Th2-related IL-5 or asthma-associated IL-32γ together with NOD1 ligand can enhance eosinophil survival and activation in vitro [11,14]. iE-DAP and MDP can synergize with IL-32γ to trigger inflammatory cytokine release through caspase 1, ERK, p38 MAPK and NF-κB signaling pathways and facilitate the adhesion between human eosinophils and bronchial epithelial cells [14]. NOD1 and NOD2 ligands stimulation also modulated the attachment of eosinophils onto the endothelial cells by downregulating L-selectin expression on human eosinophils during the early phase of allergic inflammation [9]. NOD1 and NOD2 ligands can also further induce bronchial subepithelial fibrosis, as well as elevate the levels of serum total IgE and chemokines for eosinophils and Th2 cytokines in bronchoalveolar lavage fluid of ovalbumin (OVA)-sensitized allergic asthmatic mice [9]. Intranasal stimulation of NOD2 ligand can also induce the TSLP and IL-25 expression, which disrupts the generation of Treg and blocks the tolerance towards antigens; subsequently it drives the development of eosinophils-associated airway inflammation in mice [22]. These studies indicate that bacterial infection can enhance the sensitization to inhaled allergens and augment allergic airway inflammation by directly or indirectly activating eosinophils via NLR, and through the interaction with bronchial epithelial cells in the lung to release allergic inflammatory chemokines.

### 3.3. NLR of Basophils and Bacterial Infection in Allergic Asthma

**Figure 4:**
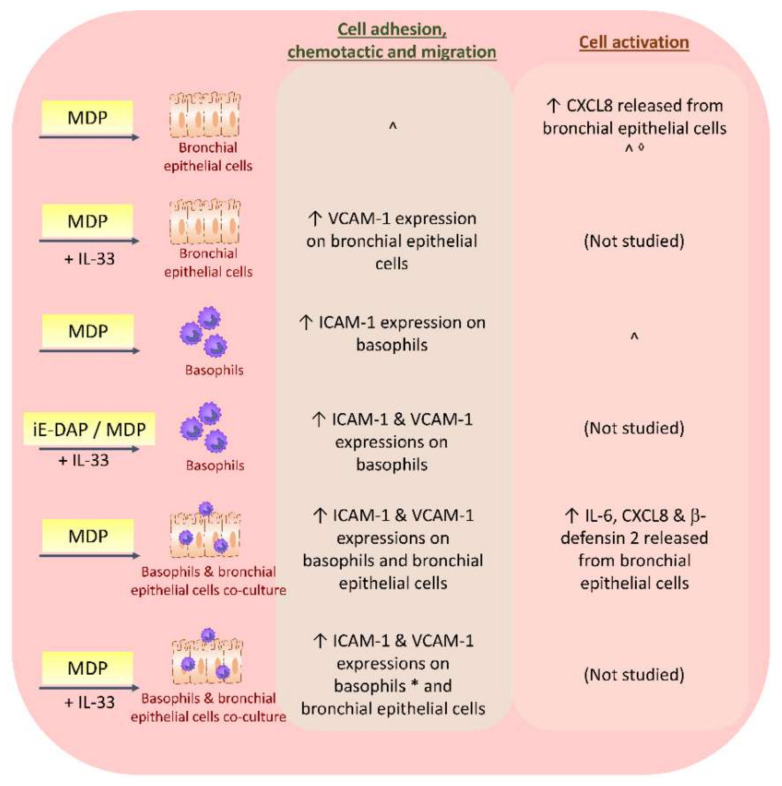
Effects of NLR ligands iE-DAP and MDP on human basophils, bronchial epithelial cells and their co-culture in vitro. NLR ligands upregulate adhesion molecules ICAM-1 and VCAM-1 in basophils and bronchial epithelial cells with or without IL-33; hence, they facilitate cell adhesion, chemotaxis and migration of leucocytes to the inflammatory lung. NLR ligands also activate bronchial epithelial cells to increase the release of pro-inflammatory cytokine IL-6 and CXCL8, as well as antimicrobial peptide β-defensin 2 in the co-culture of basophils/bronchial epithelial cells. * The increase in ICAM-1 and VCAM-1 on basophils in the co-culture system is also observed upon iE-DAP stimulation. ^ No significant difference in ICAM-1 expression or IL-6, CXCL8 or β-defensin 2 released from basophils or bronchial epithelial cells upon MDP stimulation. ^◊^ Results varied across publications.

Basophils are another type of leucocytes that produce Th2 cytokines in the late phase of allergy. NOD1 is not expressed in human basophils or basophil cell line KU812 cells. However, NOD2 is expressed in human basophils, KU812 and primary human bronchial epithelial cells [12]. NOD2 in basophils is also found to be involved in the bacterial defense and is potentially linked to the subsequent amplification of allergic airway inflammation (Figure 4). NOD2 ligand MDP can activate BEAS-2B cells and human basophils by stimulating their release of inflammatory cytokines, including Th17-polarizing IL-6, and upregulating the expression of intercellular adhesion molecule (ICAM)-1 and vascular cell adhesion molecule (VCAM)-1 in the co-culture of basophils and bronchial epithelial cells through NF-κB, c-Jun *N*-terminal kinases (JNK) and p38 MAPK pathways [12]; hence, magnifying the inflammatory response. ICAM-1 and VCAM-1 are essential for leucocytes migration to the inflammatory sites in allergy, with expressions that are positively correlated with allergic asthma severity [12,108]. MDP can also induce the release of anti-microbial peptide β-defensin 2 from the bronchial epithelial cells in the co-culture of human basophils and human bronchial epithelial cells [12]. Priming with Th2-promoting IL-33 can further enhance the stimulatory effect of MDP on human basophils [12,24], indicating that the Th2 cytokine environment, concomitantly, also promotes the MDP stimulation. The involvement of NLR in other immune effector cells recruited to the asthma pathogenesis requires further investigation.

### 3.4. NLR and Viral Infection in Allergic Asthma

NLR also play a key role in eliciting inflammatory response in allergic asthmatic patients with viral infection. Virus infection is one of the major causes of asthma exacerbation [109,110,111,112,113]. Wheezing caused by rhinovirus infection in infancy and early childhood is a robust predictor of asthma development [114]; while respiratory syncytial virus (RSV)-induced bronchiolitis is a risk factor for allergic asthma [115,116]. NLRP3 can also activate inflammasome and trigger IL-1 production upon influenza A viral stimulation [32,33,48], while NLRX1 can induce the formation of reactive oxygen species [117]. Although NOD1 and NOD2 ligands cannot trigger the release of eosinophils chemokine CCL11 from human bronchial epithelial cells [9,118,119], which are infiltrates commonly found in RSV-infected lung [120], NOD2 can normally activate the antiviral defense by recognizing the viral single-stranded RNA genome of RSV, influenza A virus and parainfluenza virus; hence, triggering the interferon-regulatory factor (IRF3) activation and the production of innate anti-viral IFN-γ [21]. However, downregulated NOD2 expression is observed in CCR3+ granulocytes of allergic asthmatic patients with or without steroid treatment when compared to healthy control subjects [121]. Impaired IFN-γ response was observed in the airway epithelial cells of asthmatic patients infected with rhinovirus [122], leading to the impaired anti-viral response. More impaired lung function was significantly observed in rhinovirus-infected children who are at higher risk of developing asthma than low risk children infected with rhinovirus [123].

## 4. Potential NLR-Related Therapy in Treatment of Allergy Based on Murine Models

As NLR and its downstream signaling pathways possess an important role in the microbial recognition in allergy development and exacerbation, they are therapeutic targets in treating allergic diseases. A recent study elucidated that dexamethasone, a conventional treatment for asthma, can alleviate allergic airway inflammation partly by inhibiting NLRP3 inflammasome and lowering IL-1β and IL-18 levels in the bronchial lavage fluid of OVA-induced asthmatic mice [103]. Sevoflurane, an agent commonly used to treat life-threatening asthma, can suppress NLRP3 expression in murine allergic airway inflammation [124]. Antitussive agent Suhuang can also improve the pulmonary function via suppressing NLRP3 inflammasome [102]. Medicinal plants such as Hibiscus noldeae, which has been traditionally used for treating asthma, were found to possess anti-inflammatory potential with NLRP3 inflammasome inhibition [125,126]. Traditional Chinese Medicine formula Yupingfeng San, identified by network pharmacology, was proven to attenuate airway inflammation via inhibiting NLRP3 inflammasome in asthmatic mice [127].

NLR-mediated mesenchymal stem cells (MSC) were shown to possess a promising therapeutic effect in ameliorating allergic diseases such as AD, allergic rhinitis and asthma [128,129,130]. Preclinical study revealed that subcutaneous administration of MDP-activated human umbilical cord blood-derived mesenchymal stem cell (UCB-MSC), which express functional NOD2, can alleviate skin inflammation and reduce the scratching behavior in HDM-induced AD murine model by reducing mast cells infiltration and degranulation in an IgE-independent manner, with cyclooxygenase-2-mediated production of PGE2 [130]. NOD inhibitors were also discovered [131,132]. It is anticipated that more anti-allergic therapy related to NLR or inflammasome can be discovered because the role and mechanism of the NLR are gradually revealed.

## 5. NLR and Trained Immunity

Although the binding between each NLR in the host and the corresponding PPRs released by the microbes and their possible interaction with allergens are still largely unknown in allergic patients, we have illustrated in the above that the recognition of the invading microbial components by the innate PRRs is more specific than we expected. Our understanding of classical immunological memory mainly falls short in relation to the adaptive immunity, which is characterized by the induction of long-lived specific memory B cells and antibody production against subsequent infections. However, the increasing discoveries on the innate-like immune memory function in plants and invertebrates prompt researchers to investigate the potential protective roles of innate immune response against reinfection [133].

As first proposed in 2011, the term trained immunity or innate immune memory, indicates that upon a long-term trigger by the exogenous and endogenous insults, the innate immunity will be primed with PAMP, and acquire the memory and ability to induce an enhanced non-specific immune response in reinfection [134]. Studies revealed that the innate immunity can be modulated by cross microbes or microbial products that the host previously encountered [133]. Previous study has also shown that the NOD2 ligand MDP can protect mice against intracellular protozoan Toxoplasma gondii infection [135], suggesting that NLR may play a role in this trained immunity.

We have highlighted in the above that microbial colonization on lesional skin of AD patients, as well as bacterial and viral infection in asthmatic patients, could elicit pro-inflammatory responses and hence exacerbation via various NLR. It has been suggested that the local trained immunity is induced in differentiated effector cells at the allergen entry site [136], i.e., the skin and epithelial barriers in AD and asthmatic patients, respectively. The notion of repeated challenges with specific microbial stimuli to induce a prolonged and an enhanced innate immune response has been suggested to exert benefits in managing inflammatory diseases. The involvement of trained immunity in the allergy protection upon microbial exposure during early childhood is also plausible. Further research elucidating the existence and mechanisms of allergic trained immunity against microbial infection via NLR is required.

## 6. Conclusions

Cytosolic NLR play a key role in regulating the development and exacerbation of AD and allergic asthma. Various gene polymorphisms in NLR identified in AD and allergic asthmatic patients may contribute to their intrinsic impairment of NLR functions in microbial recognition and host defense against bacterial and viral infection. However, whether this impairment leads to higher allergy susceptibility requires further investigation. Under the priming of allergy-associated cytokines or mediator stimulation, NLR ligands can trigger downstream signaling pathways including the NF-κB, MAPK and NLRP3 inflammasome/caspase 1. This can then lead to an enhanced production of pro-inflammatory cytokines and chemokines, and hence the recruitment of immune cells to the inflammatory sites, resulting in the exacerbation in patients. Therapy targeting these NLR and the downstream signaling players may provide new insights in treating allergic patients with microbial and viral infection-related complications. Roles of other NLR members in allergy are yet to explored. The detailed mechanisms that underpin how PAMP interacts with the NLR also requires further study.

## Figures and Tables

**Table 1:** Functional roles of NLRs in atopic dermatitis and allergic asthma.

Subfamily	*N*-Terminal Domain	NLR	Functions Associated with AD upon Activation		Functions Associated with Allergic Asthma upon Activation	
			Rodents	Human	Rodents	Human
NLRC	CARD (Caspase recruitment domain)	NOD1 (or CARD4)	Detect γ-D-glutamyl-meso-diaminopimelic acid (iE-DAP) from Gram-negative bacterial peptidoglycans [5]; Synergize with TLR signaling pathway to initiate anti-microbial response [6]	Detect iE-DAP from Gram-negative bacterial peptidoglycans [5]; Synergize with TLR signaling pathway to initiate pro-inflammatory cytokines production [7]	Expressed in mouse airway epithelial cells and some alveolar type II cells [8]; Induce bronchial subepithelial fibrosis, elevate serum levels of total IgE, chemoattractant of eosinophils and Th2 cytokines in BALF [9]; Allergen HDM activate RIP2 [10]	Expressed in human BEAS-2B cell and eosinophils [8,11], but not in basophils or KU812 cells [12]; Detect Tri-DAP to induce downstream NF-κB signaling [13]; Detect iE-DAP to induce release of pro-inflammatory cytokines and upregulate adhesion molecules and activation marker of eosinophils and BEAS-2B cells [9,14]; Downregulate L-selectin on eosinophils in co-culture [9]; Synergize with IL-5 or IL-32 γ to enhance eosinophils survival and activation, trigger cytokine release and facilitate adhesion between eosinophils and bronchial epithelial cells [11,14]; Allergen HDM activate RIP2 [10];
		NOD2 (or CARD15)	Recognize MDP from nearly all bacteria and intracellular LPS [15]; Induce exacerbation of AD-like skin inflammation in disrupted epidermal barrier [16]; Synergize with TLR signaling pathway to initiate anti-microbial response and Th1-polarizing mediators production [6,17]	Recognize muramyl dipeptide (MDP) from nearly all bacteria and intracellular LPS [15,18]; Induce inflammatory cytokines and chemokines release and increase adhesion molecules expressions in dermal fibroblasts, basophils and eosinophils [16]; Induce the release of β-defensin-2 in keratinocytes [19]; Trigger stimulation of NLRP3 inflammasome and its downstream signaling pathway [20]; Synergize with TLR signaling pathway to initiate pro-inflammatory cytokines production [7]	Recognize viral ssRNA genome of RSV, influenza virus and parainfluenza virus, and activate IRF3 and trigger IFN-γ production [21]; Allergen HDM activate RIP2 [10]; Induce bronchial subepithelial fibrosis, elevate serum levels of total IgE, chemoattractant of eosinophils and Th2 cytokines in BALF; as well as induce TSLP and IL-25 expression [9,22]; Promote Th2 polarization and susceptibility to airway inflammation [22]	Expressed in human BEAS-2B cell, primary bronchial epithelial cells, eosinophils, basophils and KU812 cells [11,12,23]; Detect MDP to induce the release of pro-inflammatory cytokines and upregulate adhesion molecules and activation marker of eosinophils and BEAS-2B cells [9,14] and in the co-culture of basophils/bronchial epithelial cells, and induce the release of β-defensin 2 from bronchial epithelial cells [12]; Downregulate L-selectin on eosinophils in co-culture [9];IL-5 or IL-32γ synergize with MDP to trigger cytokine release and facilitate adhesion between eosinophils and bronchial epithelial cells [11,14]; Allergen HDM activate RIP2 [10]; Recognize viral ssRNA genome of RSV, influenza virus and parainfluenza virus, and activate IRF3 and trigger IFN-γ production [21]; IL-33 enhance MDP stimulatory effects on basophils [12,24]
NLRP	PYD (Pyrin domain)	NLRP1 (or NALP1)	Nlrp1 is not expressed in keratinocytes * [25]; Detect anthrax lethal toxin (LT) from Bacillus anthracis to activate inflammasome * [26,27,28]	Detect MDP derived from bacterial peptidoglycan * [29];		May regulate asthma development and exacerbation [30]
		NLRP3 (or NALP3)	Sense the cellular stress induced by a wide variety of stimuli, including extracellular ATP, pore-forming toxins, uric acid crystals, bacterial RNA, whole bacteria, synthetic purine-like compounds, viral DNA, peptidoglycans, UV radiation and reactive oxygen species; and hence trigger full activation of inflammasome [3,31,32,33]; NLRP3 inflammasome can be activated by fungal components [34,35,36];Downstream players of NLRP3 inflammasome caspase 1 and IL-18 are essential for the release of TSLP in plasma and contribute to Th2 response [37,38]; Do not affect the migration of BMDC from skin to draining lymph nodes or neutrophils from blood stream to skin [1]	Sense the cellular stress induced by a wide variety of stimuli, including extracellular ATP, pore-forming toxins, uric acid crystals, bacterial RNA, whole bacteria, synthetic purine-like compounds, viral DNA, peptidoglycans, UV radiation and reactive oxygen species; and hence trigger full activation of inflammasome [3,31]; NLRP3 inflammasome can be activated by fungal components [34,35,39];NLRP3 inflammasome can induce Th2 cytokines, but the Th2 milieu downregulates caspase-1 and suppress IL-1 β release from monocytes of AD patients upon staphylococcal exotoxin α-toxin [40];Downstream players IL-18 contribute to Th2 response [41,42,43]	NLRP3 are not involved in eosinophils regulation in allergic airway inflammation [44]; Commensal bacteria aggravate allergic airway inflammation via NLRP3/IL-1β axis [45]; NLRP3 inflammasome promote Th2 cells and susceptibility to airway inflammation [46]; Caspase-1 activation dampens IL-33-dependent airway inflammation [47]; Activate inflammasome and trigger IL-1 production upon influenza A viral stimulation [32,33,48]	Not expressed in eosinophils [11]; NLRP3 inflammasome promote M2 macrophage polarization and susceptibility to airway inflammation [49]; Allergen HDM can elicit pyroptosis in bronchial epithelial cells via NLRP3 inflammasome [50]
		NLRP12	Facilitate migration of BMDC from skin to draining lymph nodes [1]; Facilitate migration of neutrophils from blood stream to skin [1]		Not involved in Th2-driven airway inflammation * [51]	
NLRX	Unrelated domain	NLRX1 (NLR family member X1)	Regulate mitochondrial anti-viral response * [52];		Produce reactive oxidative species to enhance NF-κB and JNK pathways * [52]; Modulate innate immunity against *H. pylori* infection in macrophage [53], while exposure to *H. pylori* can drive asthma protection by inducing IL-18 [54,55]	Exposure to *H. pylori* can drive asthma protection [55]

* Other potential functions related to AD/allergic asthma which are not discussed in the current review.

## Data Availability

Not applicable.
